# Recovery-phase symptoms after COVID-19 infection and anxiety levels among college students

**DOI:** 10.3389/fpsyt.2026.1921410

**Published:** 2026-09-11

**Authors:** Du Guo, Tao Liu, Yajing Xu, Luze Xie

**Affiliations:** 1 Department of Clinical Laboratory, Jinhua Maternal and Child Health Care Hospital, Jinhua, China; 2 Academy of The Zhouhuaminzu Community, Lishui University, Lishui, China; 3 College of Economics, Hangzhou Dianzi University, Hangzhou, China; 4 School of Economics, Jinan University, Guangzhou, China

**Keywords:** anxiety, college students, COVID-19 infection, mental health, recovery-phase symptoms

## Abstract

**Objective:**

This study examines the relationship between physical symptoms during the recovery phase after COVID-19 infection and anxiety levels among college students, with the aim of providing empirical evidence for post-pandemic mental health screening and health management in universities.

**Methods:**

The study used online survey data collected from college students in March 2023. After excluding straight-line responses, 394 valid questionnaires were retained. Among 372 students with a clear infection status, we examined the association between confirmed COVID-19 infection and anxiety. Among 298 students with confirmed infection, we further analyzed the associations of the number of physical symptoms during recovery, recovery time, and specific physical symptoms with anxiety.

**Results:**

Confirmed COVID-19 infection itself was not significantly associated with a higher anxiety level. Among infected students, however, the number of physical symptoms during recovery was positively associated with higher anxiety levels, whereas recovery time was not significantly associated with anxiety level. Analyses of specific symptoms showed that memory decline and chest pain were significantly associated with higher anxiety levels.

**Conclusion:**

Post-infection mental health screening in universities should not focus only on whether students have been infected. Greater attention should be paid to the burden of physical symptoms during recovery. Symptom count, cognitive changes, and chest discomfort may serve as useful supplementary indicators for post-infection follow-up and psychological risk identification.

## 1 Introduction

The COVID-19 pandemic was not only an infectious disease outbreak but also a public health event with sustained implications for youth mental health (1). College students are at a critical stage of academic development, social adaptation, and career preparation. Infection risk, changes in course arrangements, restrictions on social interaction, and concerns about family health during the pandemic may all increase psychological stress. Using data from 746,217 Chinese college students, Ma et al. (2) showed that mental health problems were highly prevalent among college students during the outbreak period. Subsequent studies suggest that this impact did not disappear completely after the pandemic entered a more normalized stage. Wang et al. (3) found that campus closure management and online learning could intensify anxiety among college students through changes in sleep patterns, reduced social connection, and lower learning effectiveness. Chen et al. (4) further noted that academic stress during the pandemic significantly increased the risk of depression among college students, while sleep quality, interpersonal relationships, and coping styles played important roles. Hoteit et al. (5) also showed that college students continued to face relatively high levels of anxiety, depression, and stress symptoms. Related studies have similarly found that college students were affected in terms of anxiety, depression, academic stress, sleep changes, and lifestyle changes during the pandemic (6–8).

As pandemic control policies changed and infection waves subsided, the question facing many college students shifted from whether they would become infected to how they would recover after infection. Nalbandian et al. (9) conceptualized persistent symptoms after acute infection as post-acute COVID-19 syndrome. Recent studies further suggest that symptoms during recovery may not only persist over time but may also affect cognitive function, emotional status, quality of life, and social functioning. In a 12-month post-infection follow-up, Ida et al. (10) reported that persistent fatigue, memory impairment, anxiety, and depressive symptoms were highly prevalent, and these manifestations were correlated with impaired physical function and diminished quality of life. A systematic review by Malioukis et al. (11) further suggested that neurological symptoms, psychosocial factors, and emotional problems such as anxiety and depression may form mutually reinforcing feedback mechanisms, thereby prolonging symptom duration and increasing the difficulty of recovery. For college students, mental health risks during this period may arise less from the fact of infection itself and more from symptoms during recovery, impaired physical functioning, and worries about sequelae.

In this study, recovery-phase symptoms refer to self-reported physical symptoms experienced during post-infection recovery. Although not defined by a specific duration, they overlap with symptoms commonly discussed in research on Long COVID and post-COVID condition. Studies of Long COVID and post-COVID condition have shown that persistent symptoms after infection have clear multisystemic features, involving respiratory, cardiovascular, neurocognitive, psychiatric, and psychological systems (12, 13). Negrut et al. (14) further noted that fatigue, dyspnea, cognitive impairment, chest pain, palpitations, muscle pain, and sleep problems are common long-term symptoms and may persist over time or present as new clinical manifestations. Walker et al. (15), using data from patients attending multiple Long COVID clinics in the United Kingdom, found that fatigue, cognitive impairment, and depressive symptoms were significantly associated with functional limitations. Fatigue was the main symptom affecting daily functioning and work ability. A longitudinal study by Hartung et al. (16) found that fatigue and cognitive deficits were among the most common and persistent sequelae following COVID-19 infection, with approximately half of affected patients still experiencing symptoms up to two years later. In addition, Szewczyk et al. (17) found that even when some patients considered themselves recovered from Long COVID, fatigue, pain, and subjective cognitive decline could persist and have long-term effects on quality of life, work, and educational activities. Among college students, reduced physical strength may affect daily activities, memory decline may interfere with classroom learning and exam preparation, and chest pain or palpitations may amplify concerns about cardiopulmonary function and possible sequelae. Recovery-phase symptoms, therefore, are not only physiological outcomes. They may also be related to learning function, quality of life, and anxiety.

The explanatory framework of this study is mainly drawn from health anxiety theory in health psychology. This perspective suggests that physical symptoms are not merely physiological signals. They may also be transformed into emotional responses through individual risk interpretation and threat appraisal. Salkovskis and Warwick (18) emphasized that threatening interpretations of bodily sensations may trigger and maintain health anxiety. Salkovskis et al. (19) further noted that bodily checking, repeated reassurance seeking, and catastrophic interpretations may reinforce the cycle of anxiety. In the psychology of pandemics, Taylor (20) emphasized that fear of infection, risk information, and bodily vigilance jointly shape psychological responses. Asmundson and Taylor (21) extended this perspective to the COVID-19 context and argued that infection-related worry, risk information, and interpretations of bodily sensations may together increase health anxiety. Subsequent studies have further found that perceived COVID-19 risk is significantly associated with anxiety, depression, and other negative emotional responses. In addition, individuals experiencing Long COVID frequently report elevated psychological distress, while depression and anxiety are associated with poorer quality of life and greater functional impairment (22, 23). Based on this perspective, this study assumes that the number and type of physical symptoms after infection are not merely indicators of physiological recovery. They may also be associated with higher anxiety levels through perceived risk, symptom interpretation, and health anxiety mechanisms.

Taken together, existing research has documented the widespread mental health problems among college students during the pandemic and has gradually clarified the multisystemic nature of persistent symptoms after COVID-19 infection. However, most studies on college student mental health have focused on the outbreak period, lockdowns, risk perception, or overall psychological distress. Less attention has been paid to symptom burden during the recovery phase after infection as a core explanatory variable. Long COVID research, by contrast, has drawn more heavily on medical or general population samples, with insufficient attention to college students’ learning function and psychological status. This study therefore shifts the focus from whether students were infected with COVID-19 to the burden of symptoms during post-infection recovery. It examines whether physical symptom burden during recovery is associated with anxiety.

Using a cross-sectional questionnaire design, this study does not make causal claims. Instead, it addresses three research objectives. First, it examines whether confirmed infection experience is statistically associated with anxiety levels among college students. Second, it analyzes the associations of recovery time and the number of recovery-phase symptoms with anxiety levels among students with confirmed infection. Third, it tests whether the results are consistent when specific recovery-phase symptoms and alternative anxiety outcomes are used. The marginal contribution of this study is to connect research on Long COVID-related symptoms with research on college student mental health. It highlights the empirical value of physical symptom burden during recovery as a potential indicator for public health follow-up and psychological risk screening in universities.

## 2 Methods

### 2.1 Data source and sample processing

The data came from an online questionnaire survey of college students conducted in March 2023. The questionnaire was distributed through the Wenjuanxing platform. Convenience sampling and snowball sampling were used to recruit respondents. The questionnaire collected demographic information, infection experience, recovery time, recovery-phase symptoms, daily exercise frequency, and anxiety-related items. A total of 427 questionnaires were initially collected. To ensure data quality, we conducted a consistency check on consecutive Likert-scale items and excluded 33 questionnaires with straight-line responses. The final analytical sample contained 394 valid questionnaires. For analyses comparing infection status, respondents who reported an uncertain infection status were excluded, leaving 372 students with a clear infected or uninfected status. Models involving recovery time and recovery-phase symptoms were restricted to 298 students with confirmed infection. Recovery time and recovery-phase symptoms have substantive meaning only among infected students. Therefore, uninfected students were not coded as having no symptoms or a recovery time of zero.

### 2.2 Variable definitions

The main dependent variable was a four-level anxiety variable coded from 1 to 4, corresponding to no anxiety, mild anxiety, moderate anxiety, and severe anxiety. As supplementary outcomes, this study also used the continuous anxiety score, any anxiety, and moderate-to-severe anxiety in robustness checks. Anxiety was assessed using seven items adapted from the core symptom domains of the seven-item Generalized Anxiety Disorder scale (GAD-7). These items covered anxiety or nervousness, worry about adverse events, difficulty relaxing, inability to control worry, excessive worry, restlessness, and irritability. The original GAD-7 was developed by Spitzer et al. (24) and has been widely used to assess generalized anxiety symptoms, with good reliability and validity reported in different populations (25). The seven anxiety items were scored on a 0 -3 scale and summed to construct the continuous anxiety score, with higher scores indicating higher anxiety. The total score ranged from 0 to 21. For the ordered logit analysis, the total score was classified into four ordered categories using the commonly used GAD-7 score ranges: 0–4, 5–9, 10–14, and 15–21. The binary robustness outcomes were defined as follows: any anxiety was coded as 1 for scores of 5 or above and 0 otherwise, while moderate-to-severe anxiety was coded as 1 for scores of 10 or above and 0 otherwise. Based on the 394 valid questionnaires, Cronbach's alpha for the seven-item measure was 0.935, indicating high internal consistency.

The core explanatory variables included confirmed infection status, recovery time, the number of physical symptoms during recovery, and specific symptoms. Confirmed infection status was constructed from the questionnaire item on COVID-19 infection experience. Students with confirmed infection were coded as 1, and uninfected students were coded as 0. Respondents who reported being uncertain were not included in the infection-status comparison. Recovery time was analyzed only among students with confirmed infection. It was coded according to respondents’ answers to the question about how long it took them to basically recover after infection, with higher values indicating a longer recovery period. The number of physical symptoms during recovery was calculated by summing six symptom categories reported after infection: reduced physical strength, long-term cough or shortness of breath, memory decline, palpitations, chest pain, and smell or taste dysfunction. The variable ranged from 0 to 6, with higher values indicating a greater burden of physical symptoms during recovery. Specific symptom variables corresponded to these six symptom categories. A symptom was coded as 1 if the respondent reported it and 0 otherwise.

Control variables included gender, grade, poverty status, only-child status, personality, pre-pandemic physical health, living alone during the pandemic, and daily exercise frequency. Female, poverty status, only-child status, introverted personality, and living alone during the pandemic were coded as 1 if the corresponding characteristic was present and 0 otherwise. Grade was included as a set of categorical dummy variables, with first-year students as the reference group. Pre-pandemic physical health and daily exercise frequency retained the original 1–5 questionnaire scales. For pre-pandemic physical health, higher values indicated better physical health. For exercise frequency, higher values indicated lower exercise frequency.

### 2.3 Statistical analysis

This study reports model specifications, sample ranges, control variables, and estimation methods. Regression results are interpreted as statistical associations between variables. The statistical analysis proceeded in four steps. First, descriptive statistics were used to describe the characteristics of students with confirmed infection and the distribution of anxiety status across groups. Second, among students with a clear infection status, ordered logit models were used to examine the relationship between COVID-19 infection and anxiety, testing whether infection experience was associated with anxiety level. Third, among students with confirmed infection, recovery time and the number of physical symptoms during recovery were analyzed in relation to anxiety level. Symptom count was then decomposed into specific symptoms. The proportional-odds assumption was assessed using the Brant test. The omnibus tests were not statistically significant for the fully adjusted model in **Table 1** (Model 2, p = 0.115), the fully adjusted model in **Table 2** (Model 2, p = 0.133), or the model in **Table 3** (p = 0.603), providing no evidence of violation of the parallel-lines assumption. Finally, robustness checks were conducted using an ordinary least squares (OLS) model for continuous anxiety score, a logit model for any anxiety, and a logit model for moderate-to-severe anxiety. HC3 robust standard errors were reported for the OLS model. All models controlled for gender, grade, poverty status, only-child status, personality, pre-pandemic physical health, living alone during the pandemic, and daily exercise frequency. Because this study used cross-sectional data, all results are interpreted as statistical associations rather than causal effects.

**Table 1:** Relationship between infection experience and anxiety among students with a clear infection status.

Variables	Model 1 (n=372)		Model 2 (n=372)	
	OR (95% CI)	P value	OR (95% CI)	P value
Infection status	0.981 (0.620, 1.552)	0.934	0.892 (0.551, 1.443)	0.641
Female			1.409 (0.940, 2.114)	0.097
Grade: second year			1.339 (0.822, 2.181)	0.241
Grade: third year			1.231 (0.729, 2.078)	0.438
Grade: fourth year			3.932 (1.809, 8.546)	<0.001
Grade: graduate student			1.113 (0.573, 2.161)	0.752
Poverty status			1.367 (0.781, 2.392)	0.274
Only child			0.853 (0.578, 1.259)	0.423
Introverted personality			1.122 (0.749, 1.681)	0.578
Pre-pandemic physical health			0.750 (0.590, 0.954)	0.019
Living alone during the pandemic			0.659 (0.296, 1.466)	0.307
Exercise frequency			0.917 (0.798, 1.055)	0.227

OR, odds ratio; CI, confidence interval. Reference categories were male, first-year students, non-poverty status, non-only child, extroverted personality, and not living alone during the pandemic. All P values are two-sided.

**Table 2:** Recovery time, symptom count, and anxiety among infected students.

Variables	Model 1 (n=298)		Model 2 (n=298)	
	OR (95% CI)	P value	OR (95% CI)	P value
Number of physical symptoms	1.221 (1.040, 1.433)	0.015	1.224 (1.035, 1.448)	0.018
Recovery time			0.983 (0.748, 1.290)	0.900
Female	1.379 (0.877, 2.168)	0.163	1.388 (0.874, 2.203)	0.165
Grade: second year	1.388 (0.804, 2.396)	0.239	1.388 (0.804, 2.395)	0.240
Grade: third year	1.488 (0.818, 2.708)	0.193	1.491 (0.819, 2.715)	0.192
Grade: fourth year	3.980 (1.565, 10.118)	0.004	4.020 (1.560, 10.360)	0.004
Grade: graduate student	0.819 (0.399, 1.681)	0.586	0.821 (0.399, 1.690)	0.593
Poverty status	1.443 (0.774, 2.687)	0.248	1.442 (0.774, 2.686)	0.249
Only child	0.721 (0.462, 1.124)	0.149	0.722 (0.463, 1.127)	0.152
Introverted personality	1.273 (0.803, 2.018)	0.305	1.271 (0.802, 2.016)	0.307
Pre-pandemic physical health	0.816 (0.625, 1.064)	0.134	0.814 (0.623, 1.064)	0.132
Living alone during the pandemic	1.070 (0.437, 2.617)	0.883	1.058 (0.426, 2.630)	0.903
Exercise frequency	1.003 (0.857, 1.173)	0.974	1.003 (0.857, 1.174)	0.967

OR, odds ratio; CI, confidence interval. Model 1 included the number of physical symptoms during recovery. Model 2 additionally adjusted for recovery time. Reference categories were male, first-year students, non-poverty status, non-only child, extroverted personality, and not living alone during the pandemic. All P values are two-sided.

**Table 3:** Specific recovery-phase symptoms and four-level anxiety.

Variables	Model 1 (n=298)	
	OR (95% CI)	P value
Recovery time after infection	0.896 (0.677, 1.186)	0.444
Reduced physical strength	0.718 (0.446, 1.158)	0.175
Long-term cough or shortness of breath	1.538 (0.976, 2.423)	0.063
Memory decline	2.039 (1.172, 3.549)	0.012
Palpitations	1.387 (0.795, 2.422)	0.250
Chest pain	2.189 (1.146, 4.179)	0.018
Smell or taste dysfunction	0.666 (0.384, 1.156)	0.149
Female	1.393 (0.868, 2.233)	0.169
Grade: second year	1.413 (0.814, 2.452)	0.219
Grade: third year	1.594 (0.864, 2.940)	0.136
Grade: fourth year	4.535 (1.725, 11.923)	0.002
Grade: graduate student	0.714 (0.342, 1.494)	0.372
Poverty status	1.432 (0.767, 2.676)	0.260
Only child	0.755 (0.480, 1.187)	0.224
Introverted personality	1.283 (0.799, 2.061)	0.302
Pre-pandemic physical health	0.849 (0.648, 1.111)	0.233
Living alone during the pandemic	1.250 (0.485, 3.222)	0.645
Exercise frequency	1.003 (0.855, 1.176)	0.971

OR, odds ratio; CI, confidence interval. Reference categories were male, first-year students, non-poverty status, non-only child, extroverted personality, not living alone during the pandemic, and absence of the corresponding symptom. All P values are two-sided.

## 3 Results

### 3.1 Sample characteristics

**Table 4** reports the characteristics of students with confirmed infection and the distribution of anxiety status across groups. Among the 298 students with confirmed infection, 185 reported some degree of anxiety, accounting for 62.1% of the sample. In terms of sample composition, the proportions of female students (55.7%), non-poverty students (87.9%), non-only children (61.4%), and introverted students (66.1%) were relatively high. In terms of anxiety distribution, the proportion of students with anxiety was relatively higher among students with poverty status, poorer pre-pandemic physical health, and a larger number of physical symptoms. Among students reporting four to six physical symptoms, 75.6% had anxiety, compared with 54.8% among those reporting no physical symptoms. Among students whose recovery time exceeded one month, all reported anxiety (100.0%). These descriptive results suggest that physical symptom burden during recovery may be associated with higher anxiety levels, but this relationship requires further examination using multivariable models.

**Table 4:** Characteristics of students with confirmed infection and distribution of anxiety status.

Characteristics	Categories	Total n (%)	No anxiety n (row %)	Anxiety n (row %)
Gender	Male	132 (44.3)	56 (42.4)	76 (57.6)
	Female	166 (55.7)	57 (34.3)	109 (65.7)
Grade	First year	87 (29.2)	31 (35.6)	56 (64.4)
	Second year	58 (19.5)	17 (29.3)	41 (70.7)
	Third year	41 (13.8)	20 (48.8)	21 (51.2)
	Fourth year	90 (30.2)	39 (43.3)	51 (56.7)
	Graduate student	22 (7.4)	6 (27.3)	16 (72.7)
Poverty status	No	262 (87.9)	106 (40.5)	156 (59.5)
	Yes	36 (12.1)	7 (19.4)	29 (80.6)
Only child	No	183 (61.4)	65 (35.5)	118 (64.5)
	Yes	115 (38.6)	48 (41.7)	67 (58.3)
Personality	Extroverted	101 (33.9)	45 (44.6)	56 (55.4)
	Introverted	197 (66.1)	68 (34.5)	129 (65.5)
Pre-pandemic physical health	Very poor	2 (0.7)	1 (50.0)	1 (50.0)
	Poor	18 (6.0)	5 (27.8)	13 (72.2)
	Fair	127 (42.6)	39 (30.7)	88 (69.3)
	Good	106 (35.6)	42 (39.6)	64 (60.4)
	Very good	45 (15.1)	26 (57.8)	19 (42.2)
Living alone during the pandemic	No	279 (93.6)	105 (37.6)	174 (62.4)
	Yes	19 (6.4)	8 (42.1)	11 (57.9)
Recovery time after infection	Within 3 days	53 (17.8)	19 (35.8)	34 (64.2)
	Within 1 week	143 (48.0)	58 (40.6)	85 (59.4)
	Within half a month	80 (26.8)	30 (37.5)	50 (62.5)
	Within 1 month	15 (5.0)	6 (40.0)	9 (60.0)
	More than 1 month	7 (2.3)	0 (0.0)	7 (100.0)
Number of recovery-phase physical symptoms	0	42 (14.1)	19 (45.2)	23 (54.8)
	1	78 (26.2)	35 (44.9)	43 (55.1)
	2	73 (24.5)	28 (38.4)	45 (61.6)
	3	64 (21.5)	21 (32.8)	43 (67.2)
	4-6	41 (13.8)	10 (24.4)	31 (75.6)
Total		298 (100.0)	113 (37.9)	185 (62.1)

Anxiety status was defined based on the total anxiety score. No anxiety was defined as a total score of 0–4, and anxiety was defined as a total score of 5–21, including mild, moderate, and severe anxiety symptoms.

### 3.2 COVID-19 infection and anxiety level

**Table 1** presents the association between infection experience and anxiety among the 372 students with a clear infection status. Model 1 included only infection status, whereas Model 2 was additionally adjusted for gender, grade, poverty status, only-child status, personality, pre-pandemic physical health, living alone during the pandemic, and exercise frequency. Infection status was not significantly associated with anxiety in either Model 1 (OR = 0.981, 95% CI: 0.620–1.552, p = 0.934) or Model 2 (OR = 0.892, 95% CI: 0.551–1.443, p = 0.641), indicating that infection experience itself was not associated with anxiety level after accounting for potential confounders. In Model 2, fourth-year students had significantly higher odds of being in a higher anxiety category than first-year students (OR = 3.932, 95% CI: 1.809–8.546, p < 0.001). Overall, infection status alone did not explain differences in anxiety levels. The subsequent analyses therefore focused on students with confirmed infection to examine whether recovery time and the number of physical symptoms during recovery were associated with anxiety.

### 3.3 Recovery time, symptom count, and anxiety

**Table 2** reports the associations of recovery time and the number of physical symptoms during recovery with anxiety among infected students. In Model 1, the number of physical symptoms during recovery was positively associated with anxiety level (OR = 1.221, 95% CI: 1.040–1.433, p = 0.015), indicating that students with a greater physical symptom burden were more likely to be in a higher anxiety category. After recovery time was added in Model 2, the association remained largely unchanged (OR = 1.224, 95% CI: 1.035–1.448, p = 0.018). Each additional recovery-phase symptom was associated with an approximately 22.4% increase in the odds of being in a higher anxiety category. By contrast, recovery time was not significantly associated with anxiety level (OR = 0.983, 95% CI: 0.748–1.290, p = 0.900), indicating no evidence of an association between recovery duration and anxiety. Overall, after adjustment for gender, grade, poverty status, only-child status, personality, pre-pandemic physical health, living alone during the pandemic, and exercise frequency, the number of physical symptoms during recovery remained significantly associated with higher anxiety levels, whereas recovery time did not. In addition to the primary explanatory variables, fourth-year students had significantly higher odds of being in a higher anxiety category than first-year students (OR = 4.020, 95% CI: 1.560–10.360, p = 0.004). This finding may partly reflect grade-specific differences in academic and career pressures.

### 3.4 Specific recovery-phase symptoms

**Table 3** further examined the associations between specific recovery-phase physical symptoms and anxiety among infected students. After adjustment for recovery time and other covariates, memory decline (OR = 2.039, 95% CI: 1.172–3.549, p = 0.012) and chest pain (OR = 2.189, 95% CI: 1.146–4.179, p = 0.018) were both significantly associated with higher anxiety levels. Students reporting these symptoms had higher odds of being in a higher anxiety category than those without the corresponding symptoms. In contrast, reduced physical strength, long-term cough or shortness of breath, palpitations, and smell or taste dysfunction were not significantly associated with anxiety. Recovery time also remained non-significant (OR = 0.896, 95% CI: 0.677–1.186, p = 0.444). However, because six symptom-specific associations were examined without adjustment for multiple comparisons, these findings should be interpreted with caution. Overall, significant associations were observed for memory decline and chest pain, whereas the other specific symptoms examined were not significantly associated with anxiety. In addition, fourth-year students had significantly higher odds of being in a higher anxiety category than first-year students (OR = 4.535, 95% CI: 1.725–11.923, p = 0.002), consistent with the findings from the previous models.

### 3.5 Robustness checks

**Table 5** presents robustness analyses using alternative specifications of the anxiety outcome. Across all three models, the number of physical symptoms during recovery showed a consistently positive association with anxiety. The association reached statistical significance in the logit model for moderate-to-severe anxiety (OR = 1.208, 95% CI: 1.001–1.457, p = 0.049), while similar positive trends were observed in the continuous anxiety score model (β = 0.587, 95% CI: −0.020 to 1.194, p = 0.059) and the any-anxiety logit model (OR = 1.198, 95% CI: 0.990–1.450, p = 0.063). Recovery time was not significantly associated with anxiety under any outcome specification. In addition, fourth-year students showed higher point estimates for anxiety than first-year students across all three alternative models, with statistically significant associations in the continuous anxiety score model (β = 4.012, 95% CI: 0.463–7.561, p = 0.028) and the moderate-to-severe anxiety logit model (OR = 4.189, 95% CI: 1.457–12.045, p = 0.008). Overall, the direction of the association between recovery-phase physical symptom burden and anxiety remained consistent across different anxiety outcome measures, supporting the robustness of the primary findings.

**Table 5:** Robustness checks using alternative anxiety outcomes.

Variables	Continuous anxiety score OLS		Any anxiety logit		Moderate-to-severe anxiety logit	
	β (95% CI)	P value	OR (95% CI)	P value	OR (95% CI)	P value
Number of physical symptoms	0.587 (-0.020, 1.194)	0.059	1.198 (0.990, 1.450)	0.063	1.208 (1.001, 1.457)	0.049
Recovery time	0.161 (-0.872, 1.194)	0.761	0.936 (0.683, 1.283)	0.683	0.911 (0.670, 1.239)	0.553
Female	1.013 (-0.540, 2.566)	0.202	1.408 (0.821, 2.414)	0.214	1.169 (0.683, 2.001)	0.570
Grade: second year	1.280 (-0.558, 3.118)	0.173	1.287 (0.681, 2.431)	0.437	1.557 (0.819, 2.958)	0.177
Grade: third year	1.310 (-0.743, 3.364)	0.212	1.602 (0.770, 3.337)	0.208	1.449 (0.712, 2.950)	0.306
Grade: fourth year	4.012 (0.463, 7.561)	0.028	2.702 (0.884, 8.260)	0.081	4.189 (1.457, 12.045)	0.008
Grade: graduate student	-0.431 (-2.606, 1.744)	0.698	0.766 (0.342, 1.714)	0.516	1.016 (0.436, 2.367)	0.971
Poverty status	1.812 (-0.335, 3.959)	0.099	2.415 (0.983, 5.933)	0.055	1.380 (0.664, 2.867)	0.388
Only child	-0.867 (-2.331, 0.596)	0.246	0.769 (0.456, 1.298)	0.326	0.613 (0.360, 1.044)	0.072
Introverted personality	0.453 (-1.129, 2.036)	0.575	1.489 (0.878, 2.525)	0.140	1.372 (0.802, 2.347)	0.249
Pre-pandemic physical health	-0.654 (-1.538, 0.229)	0.148	0.708 (0.517, 0.969)	0.031	0.804 (0.591, 1.093)	0.163
Living alone during the pandemic	0.027 (-2.988, 3.043)	0.986	0.734 (0.256, 2.108)	0.566	1.185 (0.417, 3.366)	0.750
Exercise frequency	0.045 (-0.532, 0.622)	0.879	0.942 (0.787, 1.127)	0.513	1.043 (0.874, 1.245)	0.640

β, regression coefficient; OR, odds ratio; CI, confidence interval. The continuous anxiety score was analyzed using OLS regression, and the binary anxiety outcomes were analyzed using logistic regression (n=298). Reference categories were male, first-year students, non-poverty status, non-only child, extroverted personality, and not living alone during the pandemic. All P values are two-sided.

## 4 Discussion

This study first found that confirmed COVID-19 infection itself was not significantly associated with anxiety level. This result does not deny the impact of the pandemic on anxiety among college students. Rather, it suggests that infection status may be a relatively crude indicator. Existing studies have shown that college students generally experienced high levels of anxiety, depression, and stress symptoms during the pandemic, and that this impact did not disappear completely after the pandemic entered a more normalized stage (3–5). A review by Zarowski et al. (26) further noted that infection risk, online learning, reduced social interaction, sleep problems, and economic pressure were all closely related to anxiety and psychological distress among college students. At the same time, social isolation, diminished perceived social support, and COVID-19 burnout each show significant positive correlations with elevated anxiety symptoms (27). However, these studies mainly focused on pandemic-related environmental stressors and their effects on anxiety among college students. The data used in this study were collected during a concentrated infection period after policy optimization, when most students had already been exposed to infection risk and the discriminatory power of infection status itself was reduced. This study therefore complements existing research. While previous studies have mainly examined how pandemic-related environmental stressors affected college students’ anxiety, this study further suggests that, after large-scale infection, focusing only on whether students were infected may be insufficient for identifying anxiety risk. Post-infection recovery experiences and symptom burden may be more important for understanding differences in anxiety among college students.

Among infected students, the number of physical symptoms during recovery was positively associated with anxiety level. This finding is consistent with the literature on Long COVID and post-acute COVID-19. Existing studies have shown that persistent symptoms after infection may not only last for a long time but also affect cognitive function, daily activity, quality of life, and social participation (9, 12, 13). A systematic review by Malioukis et al. (11) further suggested that neurological symptoms, psychosocial factors, and emotional problems such as anxiety and depression may form mutually reinforcing feedback mechanisms, thereby prolonging symptom duration and increasing the difficulty of recovery. Long COVID commonly presents with persistent fatigue and cognitive impairment. These symptoms have been associated with functional limitations, reduced quality of life, and lower levels of social participation (16), while recent reviews highlight their high prevalence and long-term persistence among affected individuals (14). For college students, recovery-phase symptoms may represent not only incomplete physical recovery but also difficulties in academic functioning, daily activities, and social participation. Persistent fatigue and cognitive impairment may further contribute to psychological distress and concerns about long-term health and functional recovery. According to health anxiety theory, physical symptoms are both indicators of physiological status and important cues for risk appraisal and health judgment (18, 19). As the number of recovery-phase symptoms increases, individuals may pay more attention to abnormal bodily signals and interpret them as potential health risks, thereby triggering or aggravating anxiety responses. The present study extends this medical and public health issue to the context of college student mental health. It suggests that anxiety among college students may be shaped less by infection experience itself than by persistent symptom burden after infection, along with the functional pressure and health-related worry that follow. This association should not be overstated, as the effect size was relatively limited and symptom count alone should not be understood as determining anxiety level. Moreover, because perceived health risk, symptom interpretation, and health anxiety were not directly measured, the proposed health-anxiety pathway should be regarded as an interpretive explanation rather than an empirically tested mechanism. Nevertheless, symptom count showed directionally consistent positive associations across models, indicating that it may still have value for psychological risk screening.

The nonsignificant association of recovery time also deserves discussion. In public health management, the length of time required for recovery is often treated as a health outcome. In this study, however, recovery time was not stably associated with anxiety level. One possible reason is that recovery time was measured as a relatively coarse subjective category, and students may have used different standards when judging whether they had basically recovered. Another reason is that psychological stress may be more closely related to whether current symptoms continue to interfere with study and daily life than to how many days the recovery process has lasted. However, the group with longer recovery time was small. For example, only seven students reported recovery taking more than one month. Therefore, this study cannot conclude that recovery time is completely unrelated to anxiety level. This finding is broadly consistent with a health psychology perspective: how bodily sensations are interpreted and whether they are experienced as threatening may be more closely related to anxiety responses than a single disease-course indicator (18, 19).

Among specific symptoms, memory decline and chest pain were significantly associated with higher anxiety levels. This result corresponds closely to existing research. Long COVID research has consistently found that cognitive impairment is one of the most common and long-lasting sequelae after infection, often manifested as reduced attention, memory difficulties, and cognitive slowing (14–16). A study by Takacs et al. (28) among college students and young people further found that patients with post-COVID condition performed worse in verbal working memory, divided attention, and response inhibition, and showed clear cognitive slowing. Some cognitive impairment remained present even two years after infection. For college students, memory decline may affect academic performance, exam preparation, and self-efficacy. The finding for chest pain can also be interpreted through health anxiety theory. Chest discomfort may be more easily interpreted as a sign of cardiopulmonary risk or serious sequelae, thereby increasing worry and anxiety. A scoping review by Kubrova et al. (29) on persistent post-infection chest pain noted that chest pain is a relatively common and persistent symptom of Long COVID. It may affect daily activities and quality of life, and it may also trigger concerns about possible cardiopulmonary damage. Recent studies have similarly found that patients with long-term symptoms often experience higher levels of psychological distress and functional impairment, and that physical symptoms and negative emotions such as anxiety and depression may mutually reinforce each other (10, 11, 22). Using a national sample from France, Tebeka et al. (30) further found that patients with post-COVID condition had significantly higher anxiety levels than infected individuals without post-COVID condition, and that anxiety symptoms were significantly associated with cognitive impairment, sleep problems, and overall symptom burden. These findings suggest a complex relationship between persistent physical symptoms and anxiety. Anxiety may therefore arise not only from the fact of infection itself but also from persistent physical discomfort, functional limitations, and interpretations of health risk. Because this study did not directly measure perceived health risk or health anxiety, however, this interpretation should be regarded as a theoretically plausible pathway rather than a tested mediating mechanism.

The robustness checks showed that the number of physical symptoms during recovery was not only significantly associated with four-level anxiety but also showed directionally consistent positive associations with continuous anxiety score, any anxiety, and moderate-to-severe anxiety. This result is consistent with the measurement logic of the GAD-7 described by Spitzer et al. (24), in which anxiety may be represented both as a continuous severity score and as ordered levels ranging from no anxiety to severe anxiety. The consistency of findings across different outcome measures and model forms suggests that the association between recovery-phase symptom burden and anxiety level does not depend entirely on a single dependent-variable specification.

Among grade variables, fourth-year students were more likely to be in a higher anxiety category across multiple models. Existing studies suggest that academic stress, changes in learning modes, and uncertainty about future development may increase psychological distress among college students (2–4). Using longitudinal smartphone and ecological momentary assessment data, Huckins et al. (31) found substantial changes in student mental health and behavioral patterns during the early pandemic. Barbayannis et al. (32) further found significant differences in academic stress and mental health levels across grades, suggesting that students’ developmental stage may affect their stress experiences and psychological adaptation. For senior students, physical recovery after infection may be intertwined with graduation, internships, employment, or plans for further study. Similar symptoms may therefore generate greater functional pressure and higher anxiety. However, this study did not directly measure academic pressure, employment pressure, or pressure related to further study. The grade difference should therefore not be overinterpreted. Future studies could include academic pressure, employment expectations, and social support to further explain the sources of differences in psychological risk across grades.

From a public health practice perspective, the findings support a more refined approach to campus health management. Kola et al. (33) noted that the COVID-19 pandemic created not only challenges for infectious disease control but also sustained effects on public mental health. Mental health promotion, early identification, and integrated community-level support should therefore become important components of the public health system. A scoping review by Cha and Baek (34) on Long COVID symptoms and management further emphasized that identification of persistent symptoms, multidisciplinary collaboration, and long-term follow-up are important for Long COVID management. Such management should address patients’ physical function, cognitive status, and mental health needs. In university settings, asking only whether a student has been infected may be insufficient for identifying students with higher anxiety levels. Symptom count during recovery, changes in cognitive function, and chest discomfort may serve as supplementary indicators in post-infection follow-up. Universities could integrate physical recovery follow-up with mental health screening by using a brief questionnaire two to four weeks after infection. Such a questionnaire could include symptom count, the degree to which learning function is affected, health-related worry, and anxiety level. For students with multiple symptoms, marked memory decline, or persistent chest pain, universities could provide referral pathways for medical re-examination, psychological counseling, and academic support.

## 5 Conclusion

Based on questionnaire data from college students, this study systematically examined the relationships among COVID-19 infection experience, recovery-phase symptoms, and anxiety levels. The results showed that confirmed COVID-19 infection itself was not significantly associated with anxiety level, whereas the number of physical symptoms during recovery was significantly and positively associated with anxiety. Further analysis showed that not all recovery-phase symptoms were associated with anxiety. Memory decline and chest pain were significantly associated with higher anxiety levels. Robustness checks indicated that these conclusions remained consistent across alternative anxiety outcomes and model specifications.

The findings suggest that, compared with infection experience itself, post-infection recovery experiences may better explain differences in mental health among college students. Physical symptom burden during recovery is not only a reflection of physiological recovery but may also be closely related to psychological adaptation. Therefore, post-pandemic mental health management in universities should not focus only on whether students have been infected. Greater attention should be paid to physical recovery after infection and changes in learning function.

Based on these findings, universities should combine post-infection health follow-up with mental health screening. Routine health monitoring could include recovery-phase symptoms, changes in cognitive function, and the extent to which learning is affected. Students with multiple symptoms or obvious functional impairment during recovery should receive more timely psychological support, medical consultation, and academic assistance.

This study has several limitations. First, the cross-sectional design does not establish temporal order or causality, and the observed associations between recovery-phase symptoms and anxiety may be bidirectional. Second, the sample was obtained through convenience sampling and snowball sampling, which limits representativeness. The external generalizability of the findings should therefore be interpreted with caution. Third, although the models adjusted for a range of observed covariates, potential confounders could not be fully captured. Factors such as prior mental health history, COVID-19 severity, vaccination status, time since infection, social support, and academic pressures were not included in the survey, and residual confounding therefore cannot be excluded. Finally, this study did not directly measure psychological mechanism variables such as perceived health risk or illness-related worry. It therefore cannot further reveal the specific pathways through which recovery-phase symptoms may be associated with anxiety levels. Future research could combine longitudinal follow-up data with more comprehensive clinical, psychosocial, and psychological measures to further examine the dynamic relationship between physical recovery and psychological adaptation after infection.

## Data Availability

The raw data supporting the conclusions of this article will be made available by the authors, without undue reservation.
